# Low Yield of Hepatitis C Infection in an Outreach Screening Program in Harris County, Texas

**DOI:** 10.1093/ofid/ofaa191

**Published:** 2020-06-02

**Authors:** Hyun-seok Kim, Rosalia Guerrero, Shane W Reader, Maria Daheri, Maya Balakrishnan, Catherine L Troisi, Hashem B El-Serag, Aaron P Thrift

**Affiliations:** 1 Section of Gastroenterology and Hepatology, Department of Medicine, Baylor College of Medicine, Houston, Texas, USA; 2 Management, Policy and Community Health, UTHealth School of Public Health, Houston, Texas, USA; 3 Section of Epidemiology and Population Sciences, Department of Medicine, Baylor College of Medicine, Houston, Texas, USA; 4 Harris Health System, Houston, Texas, USA; 5 Dan L. Duncan Comprehensive Cancer Center, Baylor College of Medicine, Houston, Texas, USA

**Keywords:** HCV infection, prevention, screening

## Abstract

A community outreach hepatitis C virus (HCV) infection screening program provided low yield of detecting HCV-infected patients, linking them to our hepatology clinic for treatment. Our data underscore that most of the yield was related to addiction centers and birth cohort; these groups should be targeted by future interventions.

Hepatitis C virus (HCV) infection prevalence in Texas is 23% higher than the average US prevalence at approximately 2.06% [1, 2]. Hepatitis C virus is historically the most common cause of hepatocellular carcinoma (HCC) in the United States, and Texas has the highest incidence rates of HCC among all 50 United States [1].

In 2014, the World Health Organization (WHO) set a goal to reduce by 2030 chronic viral hepatitis incidence by 90% and hepatitis-related mortality by 65%[3]. Diagnosis and awareness of infection is the first essential step towards achieving this goal. However, according to the National Health and Nutrition Examination Survey (NHANES) 2013–2016, only 55.6% of persons in the United States with HCV infection are aware of their infection status [4].

Identifying people at risk for HCV infection is important because the incidence of HCC is preventable or curable by early diagnosis and proper management. Being the most populous county in Houston (4.3 million residents), Harris County, Houston, Texas has an HCC incidence rate of 11.8/100 000, which is 1.5 times that of the entire US population. In addition, Harris County has higher HCV prevalence rates than many areas of Texas [5]. However, the identification of people at risk for viral liver disease is a challenge because those who are most at risk often do not seek regular medical care [6]. To increase awareness of the disease and link infected people to appropriate treatment, community outreach and screening programs supported by the Cancer Prevention and Research Institute of Texas were conducted in Harris County from December 2017 to February 2019. For the current analysis, we examined the yield of diagnosing and linkage to care of new positive HCV cases in this program.

## METHODS

From December 2017 to February 2019, we held educational seminars and provided printed materials to residents about viral hepatitis (risk factors, consequences, and treatment) at health fairs, community centers, addiction centers, and churches in Harris County. We used neighborhood-level information and Geographic Information Systems mapping to identify high-risk and underserved subareas in Harris County for outreach and education. Within these areas, we prioritized attending events and community settings that were more likely to host high‐risk groups, including baby boomers (adults born between 1945 and 1965) or minority (Asians, African Americans, and Hispanics) and low‐income (based on the Department of Health and Human Services poverty guidelines [7]) residents, as well as drug rehabilitation centers. The healthcare providers present at the event included physicians and nurse practitioners who had expert knowledge of viral hepatitis and liver disease. Translator service was available in English, Spanish, Vietnamese, and Chinese. We collected self-reported demographic information (age, gender, race/ethnicity) and insurance status (private, Medicare, Medicaid, or uninsured) from all participants. Before analysis, using the available information (including full name), we reviewed the patient list for duplicates. However, none were identified in our cohort.

We carried out HCV screenings using the OraQuick rapid antibody HCV test [8]. We did not distinguish between acute and chronic infections. Because OraQuick rapid antibody HCV test is based on detecting antibody, those who tested positive were most likely to have chronic (or resolved) infection. Individuals who tested HCV positive were provided with additional information and referred to Harris Health System (a publically funded safety-net healthcare system) patient navigators for confirmatory ribonucleic acid (RNA) testing and appropriate follow-up care. We followed those with positive HCV antibody test to investigate how many of these patients showed up at our hepatology clinic, received HCV antiviral therapy, and achieved sustained virologic response (SVR) based on the AASLD guidelines [9]. The Institutional Review Board at the University of Texas determined that our project does not qualify as human subject research (Reference number 148408).

## RESULTS

We screened 931 individuals through outreach programs at health fairs (47.0%), community centers (33.1%), addiction centers (11.2%), and churches (8.7%). The average age was 51.4 years old and only 39.0% were baby boomers aged between 50 and 70 years. Most were female (77.1%), and 47.7% of screened participants self-identified as Hispanic with 17.2% white, 16.5% Asian, 15.8% African American, and 2.7% other. Most participants were uninsured (79.3%) followed by Medicare insurance plan (13.5%) and private (5.9%).

Overall, only 12 of 931 (1.3%) were screened HCV positive (via the OraQuick rapid antibody HCV test). More than 80% were uninsured (N = 10). Nine were aged between 50 and 70 years, and 2 individuals born in the 1980s were screened HCV positive at residential addiction counseling facilities. Prevalence was the highest among people tested in addiction centers (8.3%, 8 of 96) followed by age group between 50 and 70 years (2.5%, 9 of 362). Eight of the 12 underwent HCV RNA testing at Harris Health System, 5 of whom were confirmed positive for HCV infection. These confirmatory tests were done without requiring a clinic appointment. Four individuals of the 12 positive for anti-HCV testing did not have a follow-up HCV RNA test despite follow-up phone calls and letters. Therefore, the prevalence of confirmed HCV ranged from 0.5% to 1.0% depending on whether the untested individuals were assumed negative or positive for HCV RNA. The 5 confirmed with HCV infection were referred to the Harris Health System hepatology clinic for treatment; however, only 3 attended their scheduled appointment and received antiviral treatment, and the other 2 did not respond to our letters or calls for an appointment. After treatment, 1 individual achieved SVR, whereas 2 participants did not attend their follow-up appointments after receiving prescription for antiviral treatment and so their status is unknown.

## DISCUSSION

Our community outreach viral hepatitis education and screening programs in Harris County, Texas showed 0.5%–1.0% HCV prevalence, which was lower than population-based estimates (approximately 2%–3%) [5]. Low linkage to care was achieved for those testing positive. This finding could reflect a misalignment between our community education targets and the high HCV risk population. Health fairs and community centers are high-traffic targets, but individuals in these facilities may be less likely to engage in the risk behaviors that often precede HCV infection. In addition, most individuals tested in our program were not in the high-risk demographic (eg, baby boomers).

To achieve the WHO’s viral hepatitis elimination goal, diagnosing 90% of HCV-infected people and treating 80% of all eligible people are required [3]. However, the incidence of HCV cases continues to increase in the United States. The Centers for Disease Control and Prevention reported that HCV incidence has almost tripled over the last 5 years, reaching a 15-year high, at 41 200 new cases in 2016 [10]. It is assumed that injection drug use among persons aged 20–39 years represents the primary source of new HCV infections in the United States. Furthermore, community residents’ poor knowledge of viral hepatitis and access to routine medical care remain big barriers for screening. A recent NHANES study suggests that HCV awareness was the lowest in Hispanics and Asians and foreign-born persons who lived below the federal poverty level and among those with low education levels [4].

Our results reflect the difficulty of broad-scale interventions that target all individuals at risk for chronic viral hepatitis simultaneously. Eckman et al [11] recently published a decision modeling that a universal 1-time HCV screening of adults 18 years or older in the United States was cost effective compared with both no screening and 1-time baby boomer screening if the prevalence of HCV antibody positivity was greater than 0.07% in the general population. This is in line with the recent update on the US Preventive Services HCV screening guidelines that recommends screening for HCV infection in adults ages 18 to 79 years [12]. As our study shows, outreach community HCV screenings are expensive and may not be the best cost-effective method for achieving this vision. It is also possible that opportunistic HCV screening in healthcare settings, such as addiction centers, primary care, emergency rooms, and elective endoscopy, is a more efficient and cost-effective approach [13].

## CONCLUSIONS

In conclusion, our large-scale community screening program had low yield of identifying HCV-infected patients and linking those infected patients to appropriate treatment for SVR. Most of the yield was related to addiction centers and birth cohort; these groups need to be targeted by future interventions.
